# Prevalence of homosexual and bisexual orientation in patients with borderline personality disorder and associated factors – a systematic review and meta-analysis

**DOI:** 10.3389/fpsyt.2024.1490157

**Published:** 2024-11-21

**Authors:** Ying Chi Camille Shu, Ka To Lau, Cyrus Su Hui Ho

**Affiliations:** ^1^ Faculty of Medicine, The Chinese University of Hong Kong, Hong Kong, Hong Kong SAR, China; ^2^ Department of Psychological Medicine, Yong Loo Lin School of Medicine, National University of Singapore, Singapore, Singapore

**Keywords:** meta-analysis, borderline personality disorder, sexual orientation, homosexuality, bisexuality

## Abstract

**Introduction:**

This meta-analysis aimed to quantitatively evaluate the association between homosexual and bisexual orientation and borderline personality disorder (BPD), including factors contributing to the association and clinical outcomes of homosexual and bisexual patients with BPD.

**Methods:**

We systematically searched PUBMED, PsycINFO, Cochrane Library, MEDLINE, EMBASE and Web of Science for cross-sectional or cohort studies comparing the prevalence of homosexual and bisexual orientation amongst patients with BPD and controls.

**Results:**

Our search identified 7 eligible studies, with a total sample case of 636 subjects with BPD and 535 subjects without BPD. Patients with BPD had a significantly higher likelihood of homosexual and bisexual orientation (Risk ratio [RR] 3.39, 95%CI 1.88-6.12) with a pooled prevalence of 28% (95% CI 0.24-0.31; I2 73%; 7 studies, 1171 participants). Subgroup analyses validated that BPD was independently associated with higher prevalence of both homosexual (RR 8.51, 95% CI 3.36-21.54) and bisexual orientation (RR 3.82, 95% CI 1.81-8.04), but no gender difference was yielded. Childhood sexual abuse (CSA) was associated with the development of homosexual and bisexual orientation in patients with BPD.

**Discussion:**

Poorer clinical outcomes, including physical and mental health, were associated with BPD status. Further studies are necessary to evaluate the feasibility and efficacy of sexual minority-specific treatment for these patients.

**Systematic review registration:**

https://www.crd.york.ac.uk/prospero/display_record.php?RecordID=538356, identifier CRD42024538356.

## Introduction

Borderline personality disorder (BPD) is characterised by pervasive emotional instability, impulsivity and difficult interpersonal relationships. These features predispose to repeated self-injury or suicidal attempts, requiring more frequent hospital admissions than other major psychiatric disorders (1). The prevalence of BPD in the community setting was estimated to be 1% according to previous studies (2). In psychiatric inpatient and outpatient clinics, the prevalence was 22% and 12% respectively (2). Patients with BPD not only more readily suffer from comorbid psychiatric disorders, including affective disorders, sleep disorders and substance use disorder, but physical comorbidities are also more common amongst patients with BPD (3). The aetiology of BPD is multifactorial, with childhood adversity, especially emotional abuse or neglect, being a significant risk factor (4).

Identity disturbance is one of the diagnostic criteria of BPD and refers to a markedly and persistently unstable self-image. Identity disturbance in BPD is likely a multifaceted psychological construct rather than a single entity. Wilkinson-Ryan et al. elicited four major identity disturbance factors: role absorption, painful incoherence, objective incoherence and lack of commitment (5). Painful incoherence, defined as a subjective sense of incoherence, was the most critical factor distinguishing BPD from other personality disorders.

On the other hand, identity disturbance is also a salient feature in the psychosexual development of the homosexual and bisexual population. Cass’s model of homosexual identity development highlighted a 6-stage process beginning with identity confusion as an individual questions his/her sexual orientation (6). Another proposed model for lesbian identity development shared a resemblance with Cass’s model, describing a sense of incongruence that lesbians might experience as they navigate their sexual orientation through interactions with the community (7). Whilst the sequentiality of these multi-stage models limited their applicability in contemporary settings, they underscored the importance of identity formation in homosexual and bisexual individuals and the likelihood of identity disturbance amidst the process.

Identity disturbance may, therefore, be a shared experience between patients with BPD and the homosexual and bisexual population. This raises suspicion for an increased prevalence of homosexual and bisexual orientation amongst patients with BPD and vice versa. This notion was supported by a previous study revealing an association between sexual minority and BPD features in a community sample of adolescents (8). Another study examining the prevalence of BPD in sexual minorities yielded similar findings, and their results derived from a national database dismissed the possibility of provider bias leading to the observed disparity (9). Instead, their findings alluded to the possibility of transdiagnostic psychopathologies unique to sexual minorities.

Homosexual and bisexual patients with BPD describe a subgroup of patients with specific therapeutic challenges. Firstly, homosexual and bisexual patients with BPD may encounter more discrimination and relationship difficulties than their heterosexual counterparts. This, combined with heightened identity disturbance intrinsic to this population, might exacerbate manifestations of BPD traits, such as increased suicidal attempts or impulsive behaviours (9). In addition, both BPD and homosexual and bisexual orientation are independently associated with more frequent risky sexual behaviours (10). Therefore, it is clinically relevant to understand the prevalence of homosexual and bisexual orientation amongst patients with BPD to provide adequate support for this subgroup. This review aims to bridge the gap in the current literature and examine the prevalence of homosexual and bisexual orientation amongst patients with BPD compared to subjects without BPD. In addition, associated factors predisposing to the development of homosexual and bisexual orientation and subsequent clinical implications, including psychiatric comorbidities, sexual and overall health status, would be evaluated.

The primary objective of this review was to assess the prevalence of homosexual and bisexual orientation amongst patients with BPD compared with subjects without BPD. Secondary objectives included evaluation of gender differences in the prevalence of homosexual and bisexual patients with BPD, factors contributing to homosexual and bisexual orientation amongst patients with BPD and the differences in clinical outcomes between heterosexual and homosexual and bisexual patients with BPD.

## Methods

This study followed the Preferred Reporting Items for Systematic Reviews and Meta-Analyses (PRISMA) guidelines and was registered with PROSPERO, CRD42024538356 (11). Institutional review board review and approval was not required for this study.

### Eligibility criteria

We included any published cross-sectional or longitudinal studies investigating the prevalence of homosexual and bisexual orientation amongst BPD. Sexual orientation was defined as any self-reported or measured patterns of romantic or sexual attraction, including any relevant sexual experience. Subjects with gender dysphoria or paraphilia were not included in this review. For study outcomes, studies were only included if any validated battery was utilised to diagnose BPD. This included the Revised Diagnostic Interview for Borderlines (12), the Diagnostic and Statistical Manual of Mental Disorders Studies or other validated batteries of choice (13). Studies must meet all of the above criteria to be considered. For the study population, studies recruiting patients with BPD with comorbid psychiatric diagnoses were not excluded, given the inherently high prevalence of concomitant psychiatric conditions in this patient population (14).

Studies meeting any one of the below criteria were excluded: (i) case controls, case studies or case reports, (ii) paediatric populations, (iii) study population not fulfilling diagnostic criteria of BPD or only demonstrating certain personality traits, and (iv) inclusion of transgender population.

### Information sources and search strategy

We identified eligible cross-sectional or cohort studies by searching PUBMED, PsycINFO, Cochrane Library, MEDLINE, EMBASE and Web of Science from inception to 8^th^ April 2024 (**Supplementary Table S1**). No publication language limitations were implemented. We used the following text words “borderline/impulsive pattern/personality/disorder/trait”, “BPD”, “emotionally unstable personality disorder”, “EUPD”, “emotional intensity disorder”, “EID”, “sex/bisexual/homosexual/pansexual/polysexual/asexual/gender orientation/attraction/behaviours/preference/leaning/identity” and relevant MESH or subject headings.

### Outcome definition

The primary outcome was the prevalence of homosexual and bisexual orientation amongst patients with BPD compared to subjects without BPD. We accepted the study authors’ definition of homosexual and bisexual orientations and/or sexual relationships with the same or both genders using validated and reliable measurement tools, such as the Barratt Impulsiveness Scale (BIS-11) (15), items within the Revised Diagnostic Interview for Borderlines (12), the ASHR Health and Relationships Questionnaire and the Abuse History Interview (AHI) (16, 17). Structured research questionnaires with questions exploring sexual orientation specifically designed for the studies were also accepted.

The secondary outcomes were the associated factors linked to homosexual and bisexual orientation in patients with BPD and the clinical implications, including psychiatric comorbidities, sexual and overall health status in homosexual and bisexual patients with BPD.

### Study selection and data extraction

Two independent review authors screened titles, abstracts and full texts to identify eligible studies using Covidence software (Veritas Health Innovation Ltd, Australia). The decision-making process for including studies in this systematic review was documented and shown in a PRISMA (Preferred Reporting Items for Systematic Reviews and Meta-Analyses) flow diagram (11).

Data from each included study were independently extracted using a standardised form by the two review authors using Covidence software. For each study, we extracted data on the title, authors, publication name, year of publication, language of publication, study design, setting, eligibility criteria, number of eligible participants, age and sex of the study participants, status of borderline personality disorder, sexual orientation, possible factors associated with homosexual and bisexual orientation and any reported clinical outcomes including sexual health and overall health status. Any disagreements were resolved by consultation with a third senior reviewer. Data were entered into Review Manager 5.4 (Copenhagen; The Nordic Cochrane Collaboration) by one author, and another author verified the data entry before meta-analyses.

### Risk of bias and certainty in evidence

Two review authors independently assessed the risk of bias and methodological quality for all included studies, utilising the Joanna Briggs Institute (JBI) critical appraisal checklist (18). An 8-item and 11-item checklist were used for appraising cross-sectional and cohort studies, respectively. Any “yes” was given 1 score, whilst “no” or “unclear” was given 0 score. The total score would be converted into a percentage of the maximum attainable score, where 70% was considered good quality and 50% was acceptable (19). Any disagreements were resolved by consulting a third senior reviewer. Studies were included regardless of the appraisal score.

### Statistical analysis and data synthesis

MetaXL was used for data analysis. Risk ratio (RR) and mean difference (MD) values with 95% confidence intervals (95% CI) were reported for dichotomous and continuous outcomes, respectively. The DerSimonian and Laird random-effects model was applied as clinical and methodological heterogeneity were anticipated among studies. The statistical heterogeneity was reported with the I^2^ statistic: an I^2^ of 25% was considered low heterogeneity, 50% moderate heterogeneity and 75% high heterogeneity. Double arcsine transformation was applied to raw data on prevalence. Pooled prevalence was derived using an inverse variance model.

## Results

**Figure 1:**
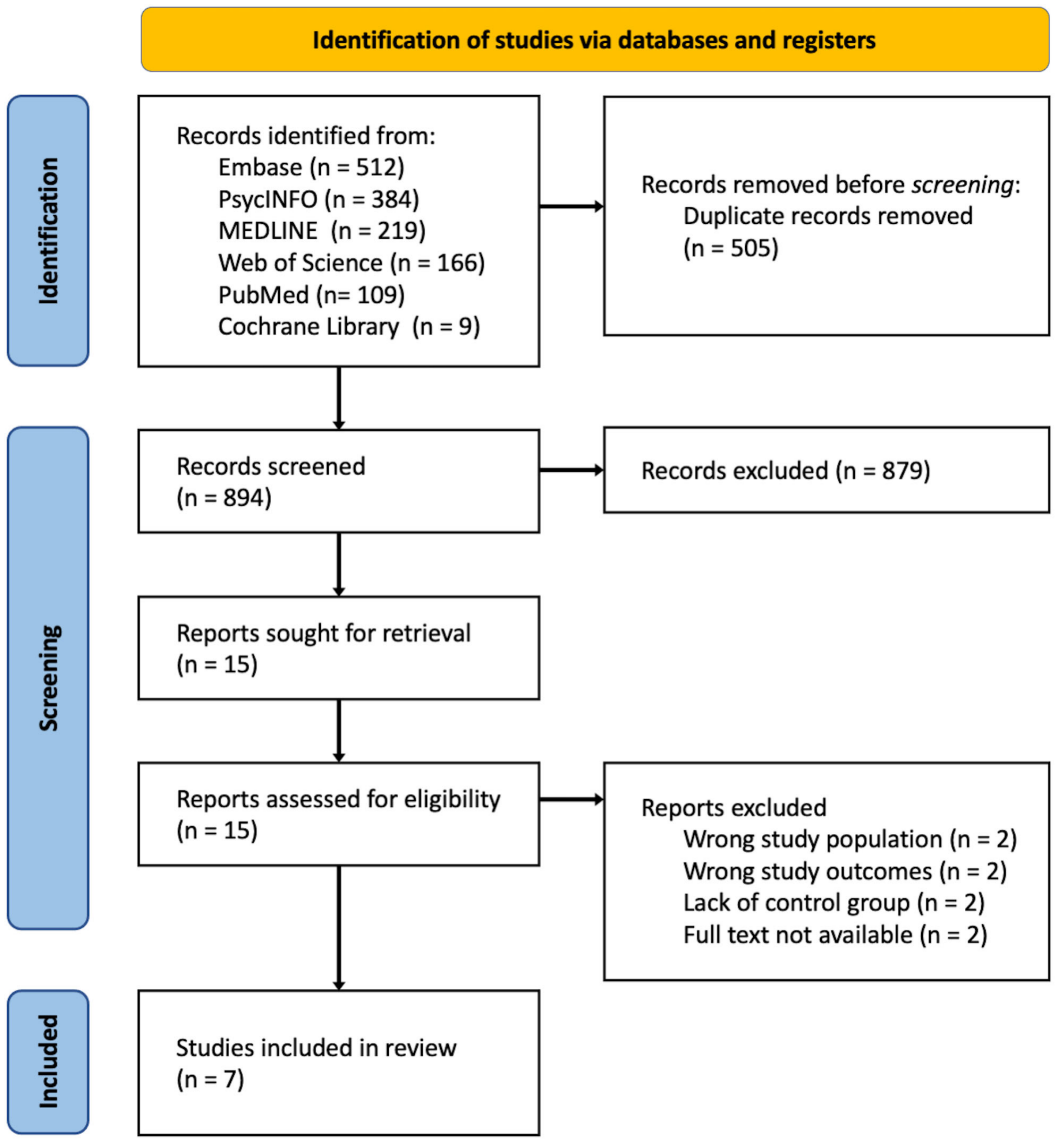
Preferred reporting items for systematic reviews and meta-analyses flow chart showing results of systematic literature search.

A systematic search identified 894 records after removing duplicates (**Figure 1**). Upon screening, 7 studies, including 6 cross-sectional studies and 1 longitudinal prospective cohort study (20–26), were included in this review. Four studies were conducted in the United States, with the remaining conducted in Australia, Canada, and Spain. A total of 1171 patients were involved in the included studies.

All the included studies recruited patients diagnosed with BPD by validated measures (**Table 1**). Most studies limited the scope to adults above 18 years old except for the studies by Thompson et al. and Zanarini et al. (24, 25). The study by Zubenko et al. did not specify any age limit for the BPD group, but the reported mean age was 24.3 ± 3.7 years (26). Three studies recruited subjects of both genders; two recruited males only, and two recruited females only. The study by Zubenko et al. limited BPD subjects to males but included both genders in the control group (26). For outcomes, five studies employed questions from validated questionnaires to assess sexual orientation, while two adopted questions from proprietary questionnaires. The studies by Paris et al. and Zubenko et al. only reported homosexuality (20, 26), whilst other studies reported both homosexuality and bisexuality.

**Table 1:** Summary of the characteristics of included studies.

Author	Study design	Population	Control	Outcome	Secondary outcome(s)
Paris et al., 1995 (20)	Cross-sectional	Patients with BPD diagnosis Age: 18-48 Gender: male only Sample size: 61	Patients with PD other than BPD Age: 18-48 Gender: male only Sample size: 60	Item 67 on the DIB-R — any sexual relationships with men over last 2 years	Semi-structured developmental interview for childhood abuse, separation or loss; PBI
Ramos et al., 2002 (21)	Cross-sectional	Psychiatric outpatients who scored ≥ 3 for BPD on the IPDE self-administered questionnaire Age: 18-65 Gender: male and female Sample size: 40	Psychiatric outpatients who scored < 3 for BPD on the IPDE self-administered questionnaire Age: 18-65 Gender: male and female Sample size: 33	Data collection protocol — sexuality and homosexual relationships	Global Activity Assessment Scale; sociodemographic and psychobiographical variables regarding childhood, interpersonal relationships, sexuality, personal and family psychiatric history
Reich and Zanarini 2008 (22)	Longitudinal prospective cohort	Psychiatric inpatients who met both DIB-R and DIPD-R criteria for BPD Age: 18-35 Gender: male and female Sample size: 290	Psychiatric inpatients who met DIPD-R criteria for ≥ 1 non-borderline axis II disorder and none of DIB-R or DIPD-R criteria for BPD Age: 18-35 Gender: male and female Sample size: 72	AHI & AHI-FUV — homosexual or bisexual orientation, same-sex relationship, change in sexual orientation, change in gender of intimate partner	Reported family history of homosexual/ bisexual orientation; AHI for childhood sexual abuse
Sansone et al., 2011 (23)	Cross-sectional	Psychiatric inpatients who exceeded cut-off scores on both PDQ-4 & SHI Age: ≥ 18 Gender: female only Sample size: 70	Psychiatric inpatients who did not exceed cut-off scores on both PDQ-4 & SHI Age: ≥ 18 Gender: female only Sample size: 56	Research booklet — homosexual experience	Other sexual behaviors including number of sexual partners, rape experience, coerced sex etc.
Thompson et al., 2019 (24)	Cross-sectional	Patients who met ≥3 DSM-IV/V criteria for BPD Age: 15-24 Gender: female only Sample size: 50	Nationally representative sample Age: 16-24 Gender: female only Sample size: 204	ASHR Health and Relationships Questionnaire — sexual health and behaviour	ASHR Health and Relationships Questionnaire for general health, substance use; Kessler Psychological Distress Scale 6-item
Zanarini et al., 2021 (25)	Cross-sectional	Adolescents who met both DIB-R and DSM-IV criteria for BPD Age: 13-17 Gender: male and female Sample size: 104	Adolescents who never met any criteria for axis I disorder or BPD Age: 13-17 Gender: male and female Sample size: 60	Adolescent versions of AHI and BIS — sexual orientation	Adolescent versions of AHI and BIS for dating history and gender of dating partners
Zubenko et al., 1987 (26)	Cross-sectional	Patients who met DSM-III criteria for BPD Age: unspecified Gender: male only Sample size: 21	Patients who met DSM-III criteria for major depression but not BPD Age: < 35 Gender: male and female Sample size: 50	Homosexual-heterosexual rating scale of Kinsey et al.	

BPD, borderline personality disorder; PBI, Parental Bonding Index; PDQ-4, Borderline Personality Scale of the Personality Diagnostic Questionnaire-4; SHI, Self-Harm Inventory; DIB-R, Revised Diagnostic Interview for Borderlines; DIPD-R, Diagnostic Interview for DSM-III-R Personality Disorders; AHI, Abuse History Interview; BIS, Background Information Schedule; IPDE self-administered questionnaire, International Personality Disorders Examination self-administered questionnaire.

### Risk of bias

The risk of bias assessment using the Joanna Briggs Institute critical appraisal tools revealed that all studies had an acceptable level of bias (**Table 2**). 4 out of 7 studies were considered good quality with a percentage JBI score above 70%. Most studies identified confounding factors, including demographic factors such as age, race and sex, but some studies did not report such baseline characteristics. Regarding strategies to deal with the confounders, some studies utilised matching within the study design, but none performed multivariate regression analysis. An issue with outcome measurement was also noted. As the outcome of sexual orientation is intrinsically difficult to assess objectively, most studies relied on self-reported scales, most of which were validated questionnaires or semi-structured interview questions.

**Table 2:** Summary of Joanna Briggs Institute Critical Appraisal Checklist.

Author	Q1	Q2	Q3	Q4	Q5	Q6	Q7	Q8	Q9	Q10	Q11	Total	%
Paris et al., 1995 (20)	Y	Y	Y	Y	Y	N	U	Y	/	/	/	6/8	75.0%
Ramos et al., 2002 (21)	Y	N	Y	Y	Y	N	N	Y	/	/	/	5/8	62.5%
Reich and Zanarini 2008 (22)	Y	Y	Y	Y	N	U	Y	Y	Y	N	Y	8/11	72.7%
Sansone et al., 2011 (23)	Y	Y	Y	Y	N	N	N	Y	/	/	/	5/8	62.5%
Thompson et al., 2019 (24)	U	Y	Y	Y	Y	Y	Y	Y	/	/	/	7/8	87.5%
Zanarini et al., 2021 (25)	Y	Y	Y	Y	Y	N	Y	Y	/	/	/	7/8	87.5%
Zubenko et al., 1987 (26)	N	Y	Y	Y	N	N	Y	Y	/	/	/	5/8	62.5%

Y, yes; N, no; U, unclear; /, not applicable; %, percentage of maximum attainable score.

### Homosexual and bisexual orientation in BPD

**Figure 2:**
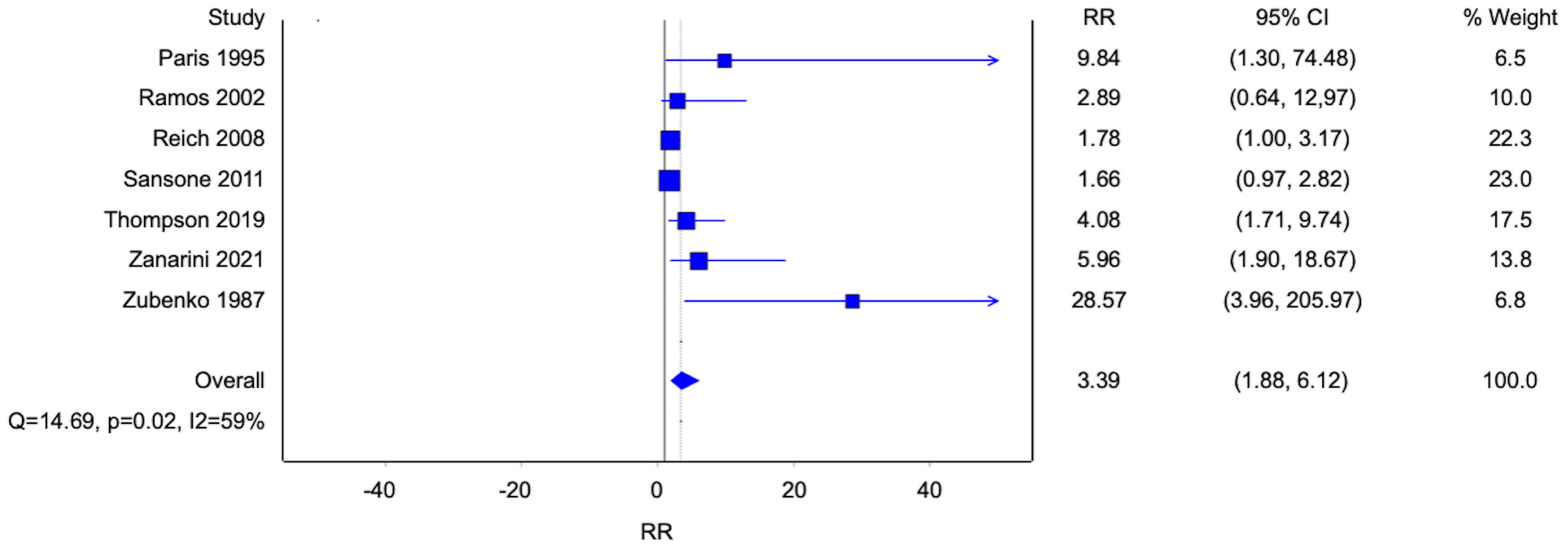
Forest plot of the risk ratio of homosexual and bisexual orientation in patients with borderline personality disorder.

Six cross-sectional studies reported the prevalence and one prospective cohort study reported the incidence of homosexual and bisexual orientation amongst patients with BPD and control groups without BPD (**Figure 2**). All studies demonstrated a higher percentage of homosexual and bisexual orientation in patients with BPD when compared to control groups. The results were statistically significant except in the studies by Ramos et al. (p=0.2) and Sansone et al. (p=0.06) (21, 23). Overall, there was a significantly higher percentage of homosexual and bisexual patients amongst patients with BPD compared to subjects without BPD. (Risk ratio [RR] 3.39, 95% confidence interval [CI] 1.88-6.12; p<0.0001; I^2^ 59%; 7 studies, 1171 participants).

**Figure 3:**
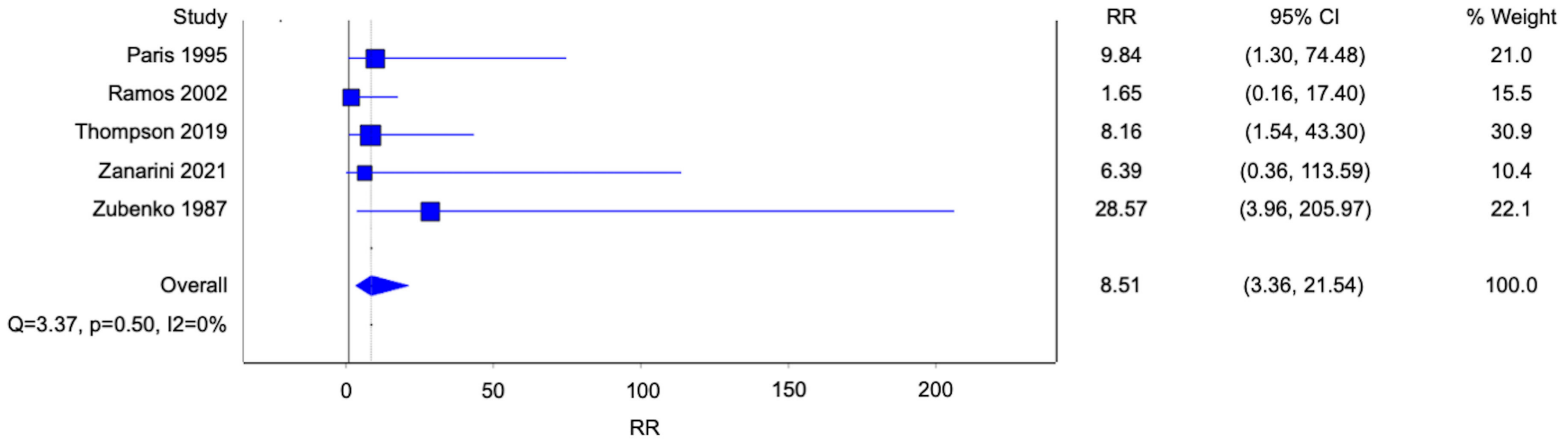
Forest plot of the risk ratio of homosexual orientation in patients with borderline personality disorder.

**Figure 4:**
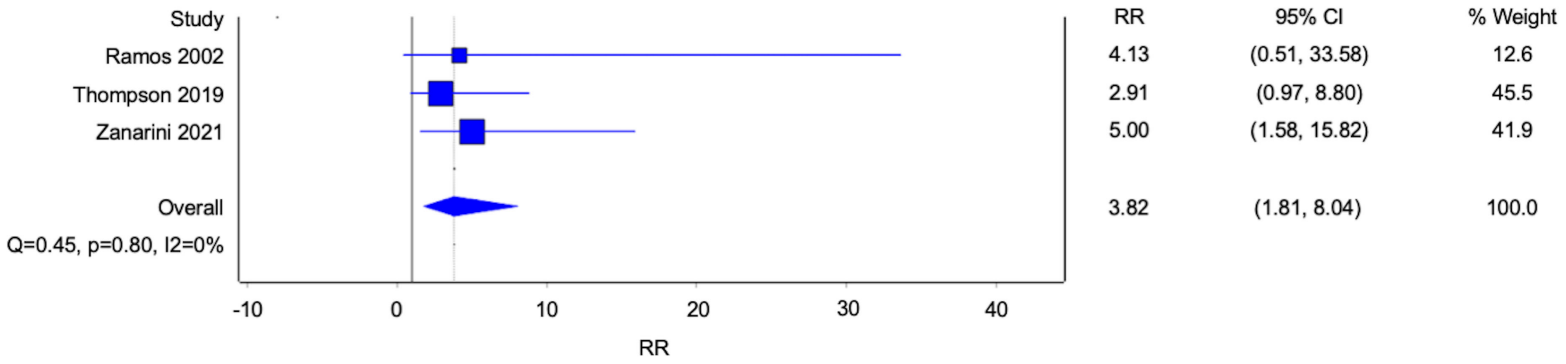
Forest plot of the risk ratio of bisexual orientation in patients with borderline personality disorder.

**Figure 5:**
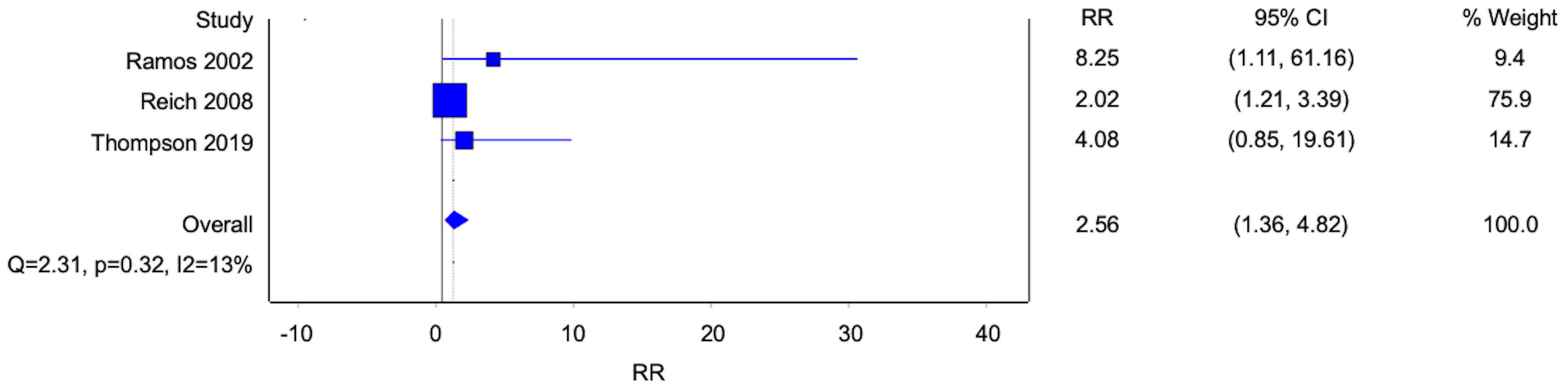
Forest plot of the risk ratio of homosexual relationships in patients with borderline personality disorder.

Five studies reported data on homosexuality, and three studies reported data on bisexuality (**Figures 3**, **4**). There were statistically significant associations between BPD and homosexuality (Risk ratio [RR] 8.51, 95% confidence interval [CI] 3.36-21.54; p<0.0001; I^2^ 0%; 5 studies, 683 participants) and bisexuality (Risk ratio [RR] 3.82, 95% confidence interval [CI] 1.81-8.04; p=0.0004; I^2^ 0%; 5 studies, 491 participants) respectively. In addition, patients with BPD were also more likely to have developed homosexual relationships (**Figure 5**) (Risk ratio [RR] 2.56, 95% confidence interval [CI] 1.36-4.82; p=0.004; I^2^ 13%; 3 studies, 689 participants).

### Prevalence of homosexual and bisexual orientation in BPD

**Figure 6:**
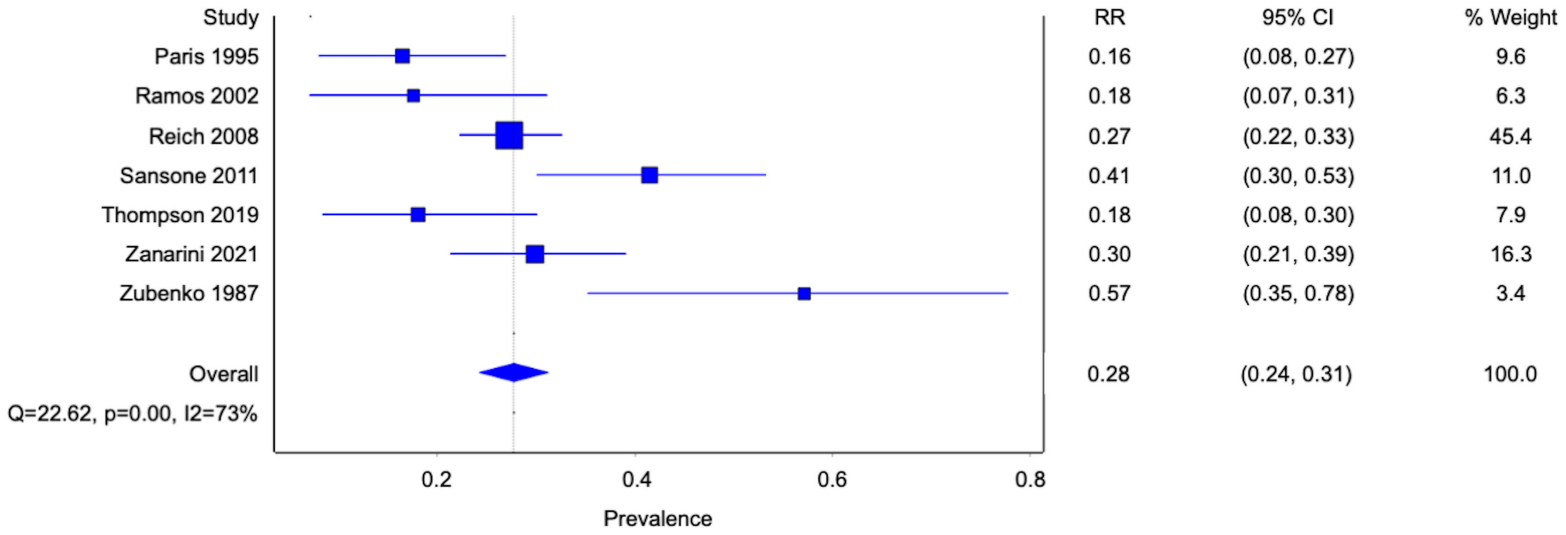
Forest plot of the pooled prevalence of homosexual and bisexual orientation in patients with borderline personality disorder.

The prevalence of homosexual and bisexual orientation amongst patients with BPD was derived from the data of each study. Reported prevalence ranged from 16-57% with moderate-high heterogeneity (**Figure 6**). The overall pooled prevalence was 28% (95% confidence interval [CI] 0.24-0.31; I^2^ 73%; 7 studies, 1171 participants). The study by Reich et al. had the most subjects and contributed to nearly half of the pooled average under the inverse variance model.

### Factor associated with homosexual and bisexual orientation: gender differences

Two studies reported the prevalence of homosexual and bisexual orientation for both genders with BPD and were used to examine for any gender differences. The two studies had contradicting results. The study by Zanarini et al. reported a much higher prevalence of homosexual and bisexual orientation in female patients with BPD than in male counterparts (25), though the statistical significance of this finding was not discussed in the original publication. The study by Reich et al. reported a slightly lower incidence amongst females (22). Overall, no statistically significant gender differences were seen. (Risk ratio [RR] 1.13, 95% confidence interval [CI] 0.37-3.45; p=0.83; I^2^ 25%; 2 studies, 394 participants) The remaining studies included only one gender or did not report gender-specific prevalence and were thus not included in the pooled effect analysis.

### Factor associated with homosexual and bisexual orientation: childhood sexual abuse

**Figure 7:**
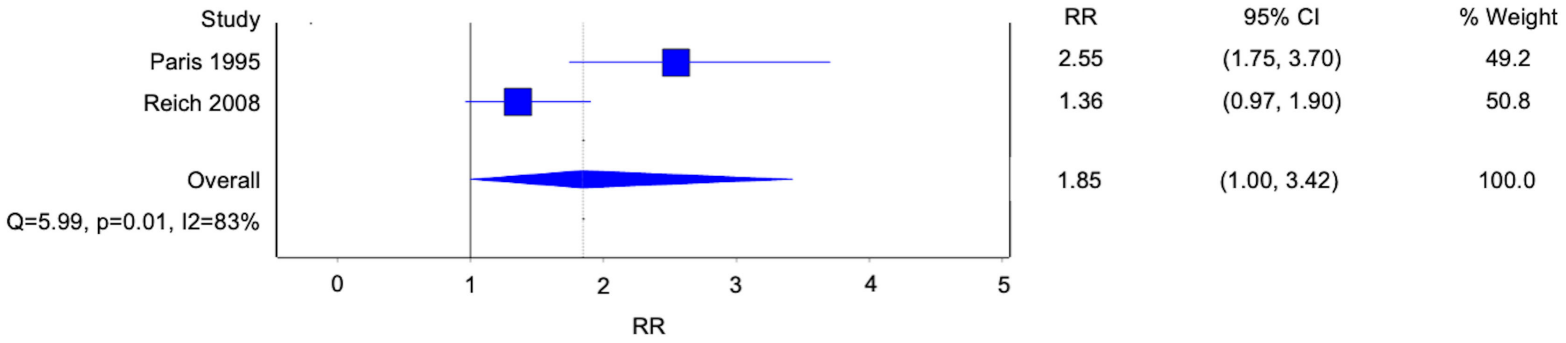
Forest plot of the risk ratio of childhood sexual abuse in patients with borderline personality disorder.

Two studies reported a history of childhood sexual abuse (CSA) as an associated factor of homosexual and bisexual orientation in patients with BPD (**Figure 7**). Both studies reported a higher likelihood of developing homosexual and bisexual orientation given a background of CSA with an overall statistically significant finding. (Risk ratio [RR] 1.85, 95% confidence interval [CI] 1.00-3.42; p=0.05; I^2^ 83%; 2 studies, 351 participants). Other studies discussing CSA as a general association with BPD without alluding to the development of homosexual and bisexual orientation were not included in this analysis.

### Factor associated with BPD: age of first sexual intercourse

Two studies reported the age of first sex as an associated factor of patients with BPD. Compared with the control group, the age of first sexual intercourse was about 1 year younger. However, this finding was not statistically significant (Weighted mean difference -0.99, 95% confidence interval [CI] -2.03-0.05; p=0.06; I^2^ 69%, 2 studies, 313 patients).

### Factor associated with BPD: number of sexual partners

Two studies compared the number of sexual partners between subjects with and without BPD. Overall, there were no statistically significant differences in the number of sexual partners between the two groups (Weighted mean difference 3.19, 95% confidence interval [CI] -1.30-7.68; p=0.16; I^2^ 69%, 2 studies, 313 patients).

### Factor associated with BPD: sexual coercion

**Figure 8:**
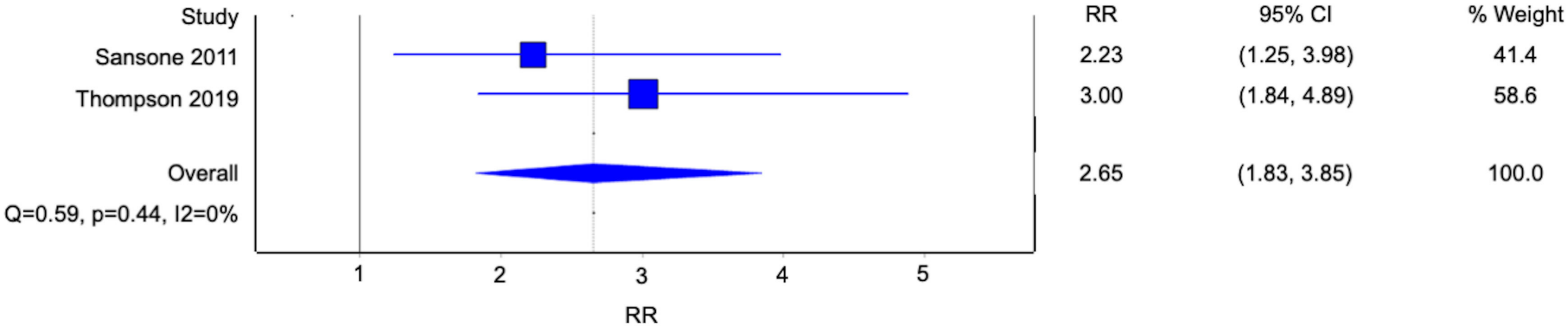
Forest plot of the risk ratio of sexual coercion in patients with borderline personality disorder.

Two studies reported a history of being coerced into sex as an associated factor of BPD (**Figure 8**). Patients with BPD were 2.65 times more likely to have been coerced into sex compared to subjects without BPD. (Risk ratio [RR] 2.65, 95% confidence interval [CI] 1.83-3.85; p<0.001; I^2^ 0%; 2 studies, 313 participants).

### Clinical outcomes

Patients with BPD had less optimal psychological health than their counterparts without BPD. Thompson et al. found that the BPD group encountered significantly greater psychological distress compared to the control group evaluated by the 6-item Kessler Psychological Distress Scale (K6) (24). This was echoed by Ramos et al., who reported that patients with BPD were 2.58 times more likely to have a history of committing suicide compared to those without (21). The hospitalisation rate of patients with BPD was reported to be 4.68 times that of subjects without BPD in the same study.

In addition to psychological well-being, overall health status was also compromised in the BPD group. Ramos et al. reported the BPD group to have poorer global functioning, reflected by a lower mean score of 70 ± 6.25 in the Global Assessment of Functioning (GAF) scale compared to subjects without BPD who scored a mean of 75 ± 9.92 (20). Thompson et al. also reported a worsening mean general health score in the BPD group compared to the control group (24). Although specific medical conditions were not explored in this study, patients with BPD were significantly more likely to smoke, consume alcohol in greater amounts and suffer concomitant substance use disorders (24). These factors combined might have contributed in part to the undermined physical health.

Concerning sexual health, studies did not reveal a higher risk of sexually transmitted diseases (STD) in patients with BPD. Sansone et al. reported an insignificant difference in the total number of times treated for STD between the BPD group and the control group (23). The study by Thompson et al. also reported similar rates of contracting most STD including HPV, genital herpes, syphilis and gonorrhoea (24). However, there was a statistically significantly higher rate of Candida/Thrush in the BPD group compared to the control group.

## Discussion

Our findings demonstrated a strong and consistent association between BPD and homosexual and bisexual orientation. Homosexuality and bisexuality were both independently associated with BPD. The phenomenon did not exhibit any gender difference. It might have arisen from a history of childhood sexual abuse, positive family history of homosexual and bisexual orientation and certain parental bonding patterns, including higher maternal or paternal control and low maternal affection (20). For secondary outcomes, BPD was associated with having more sexual partners and sexual coercion. These factors, coupled with a high prevalence of homosexual and bisexual orientation, might heighten the risks to the physical and mental well-being of patients with BPD. Previous studies reported the prevalence of homosexual and bisexual orientation in the general population to be 9.7-11.3% (27, 28). This was congruent with our findings that patients with BPD were two times more likely than subjects without BPD to be homosexual and bisexual, and a pooled prevalence of 28% among patients with BPD.

Previous evidence from a single-centre study alluded to a potential diagnostic bias leading to elevated BPD diagnosis amongst sexual minorities (29). However, our review findings pooled from multiple studies suggested a genuine association between BPD and homosexual and bisexual orientation, with CSA being a direct contributing factor. This echoed with empirical evidence that a potential causal relationship existed between CSA and homosexual orientation. In female CSA victims, the development of homosexual attraction was postulated to arise from the propensity to avoid repeating sex with males, though this model failed to account for a similar phenomenon amongst male victims (30). Nonetheless, given the higher prevalence of CSA in both BPD and homosexual and bisexual populations, it could be inferred that BPD and sexual minorities might share certain psychosocial determinants that contribute to their experiences, and leading to some overlapping areas of concern.

Homosexual and bisexual orientation in the background of BPD might thus exacerbate clinical concerns regarding BPD. Both non-suicidal self-harm (NSSH) and suicidal attempts were pervasive in patients with BPD as manifestations of their emotional dysfunction, impulsivity and hypersensitivity to interpersonal rejection (31). A population-based study further reported increased suicidal attempts in homosexual and bisexual males. It was shown that lifetime bullying and homophobic discrimination could have contributed to the increased risk of NSSH and suicidal thoughts (32, 33). These together might confer poorer prognostic outcomes for homosexual and bisexual patients with BPD. Regarding neuropsychiatric illnesses, including depression and anxiety, a previous meta-analysis with pooled data from 12 health surveys presented a doubled likelihood for homosexual and bisexual adults to report mental health complaints, especially in the bisexual group who struggled more with identity development, concealment of sexual orientation and connecting with the lesbian, gay, bisexual and transgender (LGBT) community (34). Coupling with the salient features of affective dysregulation and, thus, mood swings in BPD, homosexual and bisexual patients with BPD might have a heightened risk of suffering from an Axis I disorder.

Physical health outcomes were also compromised in homosexual and bisexual patients with BPD. For sexual health, both BPD and homosexual and bisexual orientation have been shown to significantly increase self-reported STD rates (35), rendering patients with BPD vulnerable to poor sexual health. Whilst our review did not demonstrate a significant association between BPD status and most STDs, it could be explained by the exposure to early intervention programs in the specific study sample (24). This highlighted the importance of sexual education and contraception services in holistically treating homosexual and bisexual patients with BPD. In addition, the homosexual and bisexual group exhibited a higher incidence of common chronic conditions such as hypertension, obesity and alcoholism compared to their heterosexual counterparts (36). This finding, coupled with our findings of inferior overall health in patients with BPD, underscored the importance of screening for general medical conditions among homosexual and bisexual patients with BPD.

Furthermore, treatment for BPD appeared to be less effective in the homosexual and bisexual BPD group. Dialectical behavioural therapy (DBT) is the first-line psychotherapy for patients with BPD. While both heterosexual and homosexual or bisexual patients with BPD benefited from DBT, the magnitude of improvement differed between the two groups. The homosexual and bisexual subgroup showed less change in depressive symptoms, functional impairment and other psychiatric domains (37). It was postulated that microaggressions arising from clinicians’ bias or knowledge deficit might render homosexual and bisexual patients reluctant to engage in therapy. This represented the potential challenges in treating this subset of patients with BPD and the need for specific therapeutic approaches.

Our findings highlighted the need for clinicians to recognise a high prevalence of homosexual and bisexual orientation amongst patients with BPD. Providing an accepting and non-judgmental environment with adequate safety signals would be crucial to establishing therapeutic alliance in this subgroup of patients with BPD (38). Furthermore, standard DBT models for BPD with a hierarchy of targets prioritising risky behaviours might compromise the attention to minority-related issues. Instead, specific themes like resolving identity confusion and managing cis-heterosexism should be given prominence in DBT for maximising efficacy (38). Preliminary evidence has demonstrated that psychotherapy incorporating minority stress, such as Affirmative DBT, could improve emotional regulation and depression (39). Further studies are necessary to evaluate the role of sexual minority-specific therapy in treating homosexual and bisexual patients with BPD.

Our findings paved way for future research into the interplay between sexual and gender minorities and BPD. Gender diversity is increasingly recognised in clinical settings, which may complicate the management of mental health disorders. Previous studies have shown that gender-diverse individuals often experience higher levels of borderline symptoms and suicidality (40, 41). This underscored the necessity for further investigation into how gender identities intersect with BPD across epidemiological, etiological, and clinical dimensions. Clinicians should be prepared to navigate the complexities of these associations and may benefit from adopting a more individualised, minority-specific management approach.

### Strength and limitations

Our meta-analysis was the first to systematically and quantitatively evaluate the association between BPD and homosexual and bisexual orientation. Expanding on the existing evidence, we demonstrated that BPD was independently associated with both homosexuality and bisexuality with no significant gender difference. We were also the first to demonstrate a statistical association between CSA and the development of homosexual and bisexual orientation in BPD.

Our review was limited by the inclusion of older studies of insufficient quality. Most studies were also cross-sectional and could not demonstrate a definitive causal relationship between BPD and homosexual and bisexual orientation. Moreover, patients with BPD recruited by most studies were not free of other psychiatric comorbidities. This potentially introduced confounding factors in establishing the association between BPD and homosexual and bisexual orientation.

## Conclusions

Homosexual and bisexual orientation was more prevalent amongst patients with BPD, with CSA being a contributory factor. The concurrence of BPD and homosexual and bisexual orientation elevated risks of poor physical, psychiatric and sexual health outcomes. Our review findings serve to guide the management principles targeting these specific risks and suggest the need for sexual minority-specific psychotherapy to improve clinical outcomes. Further studies should be conducted to explore the longitudinal relationship between BPD and homosexual and bisexual orientation and evaluate the specific needs of this patient subgroup.

## Data Availability

The original contributions presented in the study are included in the article/**Supplementary Material**. Further inquiries can be directed to the corresponding author.
