# Supplementary material for: Prevalence of homosexual and bisexual orientation in patients with borderline personality disorder and associated factors – a systematic review and meta-analysis
Source: Front Psychiatry. 2024 Nov 21;15:1490157. doi: 10.3389/fpsyt.2024.1490157 (PMC11617515; doi:10.3389/fpsyt.2024.1490157)
Supplement: Supplementary file 1 [file Table1.docx]

Supplemental file 1

Title: Search Strategy

OVID MEDLINE

| 1 | Borderline Personality Disorder/ |
| --- | --- |
| 2 | (borderline OR impulsive) ADJ2 (pattern* OR personality OR disorder OR trait*) OR BPD OR emotionally unstable personality disorder OR EUPD OR emotional intensity disorder OR EID |
| 3 | 1 OR 2 |
| 4 | sexuality/ or bisexuality/ or homosexuality/ or homosexuality, female/ or homosexuality, male/ |
| 5 | (sex* OR bisexual* OR homosexual* OR pansexual* OR polysexual* OR asexual* OR gender) ADJ3 (orient* OR attract* OR behavio?r* OR prefer* OR leaning OR identi*) |
| 6 | 4 OR 5 |
| 7 | 3 AND 6 |

OVID EMBASE

| 1 | borderline state/ |
| --- | --- |
| 2 | (borderline OR impulsive) ADJ2 (pattern* OR personality OR disorder OR trait*) OR BPD OR emotionally unstable personality disorder OR EUPD OR emotional intensity disorder OR EID |
| 3 | 1 OR 2 |
| 4 | sexual orientation/ or sexuality/ or bisexuality/ or homosexuality/ OR asexuality/ |
| 5 | (sex* OR bisexual* OR homosexual* OR pansexual* OR polysexual* OR asexual* OR gender) ADJ3 (orient* OR attract* OR behavio?r* OR prefer* OR leaning OR identi*) |
| 6 | 4 OR 5 |
| 7 | 3 AND 6 |

OVID APA PsycInfo

| 1 | borderline states/ OR borderline personality disorder/ |
| --- | --- |
| 2 | (borderline OR impulsive) ADJ2 (pattern* OR personality OR disorder OR trait*) OR BPD OR emotionally unstable personality disorder OR EUPD OR emotional intensity disorder OR EID |
| 3 | 1 OR 2 |
| 4 | sexuality/ or asexuality/ or pansexuality/ or sociosexual orientation/ |
| 5 | sexual orientation/ or asexuality/ or lgbtq/ or coming out/ or "homosexuality (attitudes toward)"/ or same sex marriage/ or sexual attraction/ or sexual identity/ |
| 6 | homosexuality/bisexuality/ or lgbtq/ or lesbianism/ or male homosexuality/ or sexual minority groups/ |
| 7 | (sex* OR bisexual* OR homosexual* OR pansexual* OR polysexual* OR asexual* OR gender) ADJ3 (orient* OR attract* OR behavio?r* OR prefer* OR leaning OR identi*) |
| 8 | 4 or 5 or 6 or 7 |
| 9 | 3 and 8 |

Cochrane Library

| 1 | MeSH descriptor: [Borderline Personality Disorder] explode all trees |
| --- | --- |
| 2 | (borderline OR impulsive) NEAR/2 (pattern* OR personality OR disorder OR trait*) OR BPD OR emotionally unstable personality disorder OR EUPD OR emotional intensity disorder OR EID |
| 3 | MeSH descriptor: [Sexuality] explode all trees |
| 4 | (sex* OR bisexual* OR homosexual* OR pansexual* OR polysexual* OR asexual* OR gender) NEAR/3 (orient* OR attract* OR behavio?r* OR prefer* OR leaning OR identi*) |
| 5 | #1 or #2 |
| 6 | #3 or #4 |
| 7 | #5 and #6 |

Web of Science

| (TS=((borderline OR impulsive) NEAR/2 (pattern* OR personality OR disorder OR trait*) OR BPD OR emotionally unstable personality disorder OR empd OR emotional intensity disorder OR EID)) AND TS=((sex* OR bisexual* OR homosexual* OR pansexual* OR polysexual* OR asexual* OR gender) NEAR/3 (orient* OR attract* OR behavio?r* OR prefer* OR leaning OR identi*)) |
| --- |

PUBMED

| ("borderline pattern"[tiab:~1] OR "borderline patterns"[tiab:~1] OR "borderline personality"[tiab:~1] OR "borderline disorder"[tiab:~1] OR "borderline trait"[tiab:~1] OR "borderline traits"[tiab:~1] OR "impulsive pattern"[tiab:~1] OR "impulsive patterns"[tiab:~1] OR "impulsive personality"[tiab:~1] OR "impulsive disorder"[tiab:~1] OR "impulsive trait"[tiab:~1] OR "impulsive traits"[tiab:~1] OR BPD OR "emotionally unstable personality disorder"[tiab:~1] OR empd OR "emotional intensity disorder"[tiab:~1] OR EID) AND ("sexual orientation"[tiab:~1] OR "sexual attraction"[tiab:~1] OR "sexual attractions"[tiab:~1] OR "sexual behaviour"[tiab:~1] OR "sexual behaviours"[tiab:~1] OR "sexual behavior"[tiab:~1] OR "sexual behaviors"[tiab:~1] OR "sexual preference"[tiab:~1] OR "sexual preferences"[tiab:~1] OR "sexual leaning"[tiab:~1] OR "sexual identity"[tiab:~1] OR "sexual identities"[tiab:~1] OR "bisexual orientation"[tiab:~1] OR "bisexual attraction"[tiab:~1] OR "bisexual attractions"[tiab:~1] OR "bisexual behaviour"[tiab:~1] OR "bisexual behaviours"[tiab:~1] OR "bisexual behavior"[tiab:~1] OR "bisexual behaviors"[tiab:~1] OR "bisexual preference"[tiab:~1] OR "bisexual preferences"[tiab:~1] OR "bisexual leaning"[tiab:~1] OR "bisexual identity"[tiab:~1] OR "bisexual identities"[tiab:~1] OR "homosexual orientation"[tiab:~1] OR "homosexual attraction"[tiab:~1] OR "homosexual attractions"[tiab:~1] OR "homosexual behaviour"[tiab:~1] OR "homosexual behaviours"[tiab:~1] OR "homosexual behavior"[tiab:~1] OR "homosexual behaviors"[tiab:~1] OR "homosexual preference"[tiab:~1] OR "homosexual preferences"[tiab:~1] OR "homosexual leaning"[tiab:~1] OR "homosexual identity"[tiab:~1] OR "homosexual identities"[tiab:~1] OR "pansexual orientation"[tiab:~1] OR "pansexual attraction"[tiab:~1] OR "pansexual attractions"[tiab:~1] OR "pansexual behaviour"[tiab:~1] OR "pansexual behaviours"[tiab:~1] OR "pansexual behavior"[tiab:~1] OR "pansexual behaviors"[tiab:~1] OR "pansexual preference"[tiab:~1] OR "pansexual preferences"[tiab:~1] OR "pansexual leaning"[tiab:~1] OR "pansexual identity"[tiab:~1] OR "pansexual identities"[tiab:~1] OR "polysexual orientation"[tiab:~1] OR "polysexual attraction"[tiab:~1] OR "polysexual attractions"[tiab:~1] OR "polysexual behaviour"[tiab:~1] OR "polysexual behaviours"[tiab:~1] OR "polysexual behavior"[tiab:~1] OR "polysexual behaviors"[tiab:~1] OR "polysexual preference"[tiab:~1] OR "polysexual preferences"[tiab:~1] OR "polysexual leaning"[tiab:~1] OR "polysexual identity"[tiab:~1] OR "polysexual identities"[tiab:~1] OR "asexual orientation"[tiab:~1] OR "asexual attraction"[tiab:~1] OR "asexual attractions"[tiab:~1] OR "asexual behaviour"[tiab:~1] OR "asexual behaviours"[tiab:~1] OR "asexual behavior"[tiab:~1] OR "asexual behaviors"[tiab:~1] OR "asexual preference"[tiab:~1] OR "asexual preferences"[tiab:~1] OR "asexual leaning"[tiab:~1] OR "asexual identity"[tiab:~1] OR "asexual identities"[tiab:~1] OR "gender orientation"[tiab:~1] OR "gender attraction"[tiab:~1] OR "gender attractions"[tiab:~1] OR "gender behaviour"[tiab:~1] OR "gender behaviours"[tiab:~1] OR "gender behavior"[tiab:~1] OR "gender behaviors"[tiab:~1] OR "gender preference"[tiab:~1] OR "gender preferences"[tiab:~1] OR "gender leaning"[tiab:~1] OR "gender identity"[tiab:~1] OR "gender identities"[tiab:~1]) |
| --- |
